# Comment on Choi, Y.-J.; Choi, H.-B.; O’Donnell, M. Disaster Reintegration Model: A Qualitative Analysis on Developing Korean Disaster Mental Health Support Model. *Int. J. Environ. Res. Public Health* 2018, *15*, 362

**DOI:** 10.3390/ijerph16152789

**Published:** 2019-08-05

**Authors:** Kiyoumars Allahbakhshi, Katayoun Jahangiri, Davoud Khorasani-Zavareh

**Affiliations:** 1 Department of Health in Disasters and Emergencies, School of Public Health and Safety, Shahid Beheshti University of Medical Sciences, 198353-5511 Tehran, Iran; 2 Safety Promotion and Injury Prevention Research Center, Shahid Beheshti University of Medical Sciences, 198571-1151 Tehran, Iran

**Keywords:** disaster, mental health, qualitative study, Korea

## Abstract

Recently, Choi et al. published an article in the International Journal of Environmental Research and Public Health, indicating that mental health disorders were experienced by disaster survivors in Korea. Comments were made on topics such as the participant selection, the standardization of vocabulary in disasters, choosing three different types of hazards, the type of interviews, and the language applied for interviews. This Commentary sets out to improve as much as possible the quality of the article.

Recently, a paper was published by Choi et al. in the International Journal of Environmental Research and Public Health [1], which we studied with great interest. From the appropriate choice of grounded theory, the authors tried explaining mental health disorders experienced by disaster survivors in Korea. In this paper, it was recommended that sufficient attention should be given to cultural and spiritual components, to provide appropriate mental health recovery in terms of individual and public resilience. Nevertheless, the following shortcomings must be addressed:

1. In this study, participants were selected based on snowball sampling and theoretical sampling. According to the research question, snowball sampling was used to identify participants with similar characteristics. These individuals were able to recommend new potential cases for study [2]. On the other hand, in theoretical sampling, concepts related to phenomena emerge via data [3]. As Strauss and Corbin mentioned, the purpose of this type of sampling is for “the dimensional range or varied conditions along which the properties of concepts vary” [4]. In this paper, the use of posting advertisements for the initial selection of participants created the possibility of selection bias. Thus, in the snowball sampling process, people with chronic mental health problems who did not want to participate in social activities were not included. On the other hand, there was no mention of the history of previous illnesses, which can contribute to understanding the experiences, coping strategies, and the association of these people’s health problems with disaster.
2. The results of the study showed that survivors experienced three types of disaster, including flood, fire, and road traffic incidents. The subtle note to be taken into account is that there is a need for a standardization of vocabulary, based on upstream documents, to understand and use the same concepts for disaster risk reduction. According to the United Nations International Strategy for Disaster Risk Reduction (UNISDR) terminology, there are differences between the terms “disaster”, “emergency”, and “hazard”, of which health outcomes and consequences differ greatly (Table 1) [5,6]. Therefore, it might have been better for the writers to be more careful when using the term of disaster in both the title and other sections of the article.
3. Choosing three different types of hazards at the same time can create a concern that the experiences of the exposed individuals are different.
4. Providing further explanation in the introduction and discussion about the incidence of road traffic accidents (RTAs) and floods, together with their consequences on the health system and people in Korea, contributed to the richness of the article. It also introduced readers to the current status of these disasters and emergencies in Korea.
5. Another considerable subject is the age and gender of the participants. In the methodology, the age range of the participants was mentioned as being from 20 to 64 years for adults and 65 years upward for elderly adults; however, Table 1 shows the age of people being higher than 37.
6. In this research, the type of interview was not specified. There are three types of interviews in qualitative studies: unstructured, semi-structured, and structured interviews. However, in grounded theory, the use of structured interviews is limited due to a lack of flexibility [4]. Therefore, interviews begin with open-ended questions. Then, more questions are asked based on theoretical sampling and the extracted concepts [7,8].
7. Finally, the authors needed to mention the language applied for interviews [9].

If authors would like to consider our suggested comments, the quality of their article may improve.

## Figures and Tables

**Table 1:** Definitions of hazard, emergency, and disaster based on the United Nations International Strategy for Disaster Risk Reduction (UNISDR) terminology [5,6].

Term	Definition
Hazard	A substance, phenomenon, condition, or dangerous human activity that may cause damage to humans and property, as well as the disruption of services and livelihood. Hazards have social, economic, and environmental impact. Types of hazards include hazards from natural, environmental, and technological sources, created from a variety of meteorological, geological, oceanic, hydrological, technological, and biological sources or their combination.
Emergency	Emergencies threaten situations that require urgent measures. Performing effective emergency management can prevent the event from becoming a disaster.
Disaster	A disaster means being faced with a hazard that could seriously disrupt the performance of a community. Disaster impacts may include damage to human health, in addition to property, social, economic, and environmental losses. In a disaster, the resources of the affected community are inadequate in coping with its potentially negative consequences.
